# Reflections on Failures and Human Errors

**DOI:** 10.21470/1678-9741-2023-0961

**Published:** 2023

**Authors:** Rodolfo A. Neirotti, Luiz F. Caneo

**Affiliations:** 1 Clinical Professor of Surgery and Pediatrics, Emeritus, Michigan State University, Michigan, United States of America.; 2 Pediatric Cardiac Surgery Unit, Cardiovascular Surgery Department, Instituto do Coração, Faculdade de Medicina da Universidade de São Paulo, São Paulo, SP, Brazil.

“Success is not final. Failure is not fatal. It is the courage to continue that counts.”-Winston Churchill.

Cardiac surgery is a complex system.

During our training in cardiac surgery, we learned that failures and errors were not acceptable in our practice, but after working for some time, we realized that both are inevitable and that to err is human.

Failures remind us that no one is perfect and that there is too much to know in this world. Every failure can be a step toward success if we remember that there is seldom a second opportunity to make a first good impression!

Mistakes train us to be stronger and help us to learn how to manage our emotions and realign our focus.

Furthermore, we should stay humble, hope for improvements, and be committed to lifelong learning because many benefits can be gained if we change our thoughts about how we view failures and embrace them.

This Editorial shares some views on a few aspects of practicing medicine and cardiac surgery that require understanding various facets and requirements of the specialty. We hope that the topics discussed in this article will have a positive influence in diminishing the errors and failures that cause problems, as well as other issues affecting the efforts to improve the quality of life of those in suffering.

We want to remind the readers that, in medicine, starting a practice requires understanding and remembering that the patient is the center of the practice. Therefore, we must have a strategy because the work rules are changing. A new yardstick is judging us: not just how smart we are or our expertise, but also how well we handle ourselves and each other^[^1^]^. Altogether, the critical and honest analysis of why we do what we do help us to understand the priorities of those that need help — bearing in mind that emotional intelligence can matter more than the intelligence quotients^[^1^]^.

Furthermore, a set of clear ideas and knowledge about our field and work are needed, and our practice should be based on the ethical values by which we live — keeping in mind that one of the ethical standards of the Hippocratic oath is not to harm.

The team should be committed to serve others rather than serve themselves as suggested in the Chinese philosopher Lao Tzu’s quote: “To lead the people, you must walk behind them”..

## Patient Safety Law

Because the quality of health care is often adversely influenced by errors, wrong decisions, and lack of resources, the patient’s safety and trust are diminished, which stimulates the study of the indications of high-risk procedures and the capability of those working with modern technologies in complex scenarios. Due to these concerns, a document supported by the National Academy of Medicine of Argentina was presented at the Congreso de la Nacion Argentina to discuss the need to improve their unsatisfactory results. This objective has international support and justifies the investment and the selection of the acting professionals (personal communication from Dr. Jose Navia, former president of National Academy of Medicina of Argentina, 2012-2014).

However, it is hard to believe that some people disagree with improving the quality of care for those who need it. Then, what to do with those in dissent that lost trust in what they read? — probably because we were not clear in explaining them in terms that the majority could understand. As Hannah Arendt wrote in her magnificent early work on love and how to live with fear: “Fearlessness is what love seeks.”.

Such fearlessness exists only in the complete calm that can no longer be shaken by events expected of the future... Hence the only correct tense is the present.

*Quality* is an essential and distinguishing attribute concerning something or someone. It is measurable and requires knowledge and appreciation to recognize it.

*Precision* in medicine implies a correct diagnosis plus doing what is needed the first time that can cut costs substantially. If we add the patient’s history, we end up with the following equation: quality of care = technical expertise + service quality. To achieve these humanistic demands of our profession, we need the basic authority to generate changes from the top and bottom-up involvement. *Accuracy and efficiency* are a combination for the patient’s benefit because that can cut expenses significantly.

## Definition of Errors and Failures

Determinism is a philosophical view where all events are entirely determined by previously existing causes as suggested by Lord Kelvin (1824-1907), an Irish mathematical physicist and engineer that said: “What is not defined cannot be measured. What is not measured cannot be improved. What is not improved always deteriorates.”.

*Errors are actions that are incorrect and can cause problems.* They can happen accidentally or as a result of a mistake. There are three types of errors: slips and lapses, which are unplanned or unintended actions, and mistakes, which can happen to anyone, even those who are experienced and well-trained.

Slips and lapses often occur when someone is not following their supervisor’s instructions or ignoring the correct way of doing something. This can be due to a lack of training or both. Mistakes occur when people think they are prepared but things do not work out as expected.

To avoid errors, it is important to teach based on good examples. This can help prevent unplanned or unintended actions and mistakes from happening.

*Failures are the inability to meet an expectation* — when you know something is wrong but don’t know the cause. There are three types — active failures, latent failures, and normal accidents — which are often the result of wrong plans and controlled experiences trying to figure out the cause. Again, teaching based on good examples is the key to avoiding these unpleasant mistakes.

Any successful person will admit that failures and errors happen and that we all will have a fair amount of them. Since it is your responsibility to prevent them, in case you fail, don’t get disappointed if you fail because from each failure, we can learn three valuable lessons. *One,* there was at least one reason why we failed; *two,* we could recover from that failure if we understood what happened to prevent getting things wrong again; *three,* even a single significant failure could trigger interdependences, building codes, and regulations.

## The Complete Picture of Failing

In general, slips and lapses are more basic types of errors that are related to the execution of a task, while mistakes are considered to be more complex errors that are related to the planning and execution of a task.

Most people fail not because of a lack of knowledge or talent but more often for not being persistent and giving up soon. Some people fail due to a lack of confidence and courage. Others follow the majority to be accepted even when they know what they are doing is wrong. Losers often rationalize and have multiple excuses to justify why they couldn’t go ahead. Conversely, winners might analyze, but they never excuse.

“We learn more from our failures and errors than our successes. We usually find out what does not work to correct our future efforts — learn about ourselves and get a small piece of empathy to help others who might be struggling.” - Kealy Spring, Leadership Fellow Coach^[^2^]^.

Life is complex, and errors and failures are inevitable, but we could learn more from them than from successes — then not only we should find out what doesn’t work to adjust our future attempts, but we also should learn about ourselves, gain experience time, and the necessary empathy to help others that are struggling.

“We often discover what will work by finding out what will not do — then probably those who rarely make a mistake will never make a discovery.” - Samuel Smiles.

As much as failures and errors hurt, they remain an essential part of life and are great teachers — they are helpful and necessary because we learn the greatest lessons that life can teach us through them. Moreover, they are nature’s mold that chips away all the excess — by stripping down egos as it shapes us.

More importantly, without disappointments, we would be less capable of having compassion, empathy, and kindness to fulfill our dreams.

We can find, at least, six reasons why *failures* are just as important as *success:*

1. The benefits of failures are inevitable and makes us stronger
2. Failures give us a sense of direction
3. Failures teach us values
4. Failures help us to overcome fear
5. Failures are an opportunity
6. Failures are experiences difficult to forget

In addition, failures and mistakes form our future more than success does. What you do with them makes the difference, which explains that the best lessons often come from them.

“Success is not the key to happiness. Happiness is the key to success. If you love what you are doing, you will be successful.” - Albert Schweitzer, The Humanitarian of Many Talents.

## But Why Do We Fail?

There are many causes of failures — poor leadership, lack of persistence, absence of conviction, rationalization, dismissal of past mistakes, and scarcity of discipline^[^3^]^.

We would like to emphasize four of them:

1. *Poor self-esteem:* when people lack confidence about who they are and what they can do it can make them feel incompetent, unloved, or inadequate — rather than trying to be the person they would like to be.
2. *Fatalistic attitude:* delay people from accepting responsibility for their position in life because they often attribute success and failure to luck. Conversely, those that accomplished something useful in life did it with discipline and hard work that take self-control, sacrifice, and avoiding distractions.
3. *Living by your own rules:* a teenager’s attitude that it is acceptable to break rules that people don’t like — believing they will be better than others. If not, it’s because they put themselves in a victimized/infantilized position, where the world is against them. Laws are for others, not for them; therefore, they make participating in a collective project more complicated.
  It’s a behavior noted in some places where the notion that the game can be played according to its virtues — and not customary laws — becomes in fact a source of problems that are actually solvable from certain points of view. The law must workto regulate the ends, instead of favoring one of the poles^[^4^]^.
4. *Parallel play:* is a developmental phase in early childhood where young children play independently while being in the presence of other children. When this condition occurs in an adult setting — in which each one does his/her things independently from others — we should consider it a problem. In addition, where an organization operates without a clear understanding of its governance system, allowing all parties to move forward independently, the overall quality and stability of the organization can suffer. The term “governance system” generally refers to the rules, processes, and structures that guide decision-making and ensure organizational accountability. When an organization lacks clarity around these fundamental questions, it can lead to confusion and disorder. It can be particularly damaging when the organization has no collective objectives or goals^[^5^]^.

Failures can also occur due to wrong decisions. Decision making is an intellectual process that involves knowledge, imagination, reasoning, evaluation, and judgement. It is often technical due to the narrow choices. Furthermore, in case of doubt it is important to collect information and select the best or the most suitable course of action among the available alternatives.

In medicine, wrong assignments by those in charge and communications breakdowns are common causes of errors. The decision maker should be responsible for the result, complications, and consequences!

## Importance of Failures and Errors

What do we learn from failures? From failures, we learn *resilience,* that can help us in other situations too — the capacity or not to recover quickly from difficulties is an important life skill to build. Learning from failure can be challenging, but it becomes easier when an individual is determined to overcome their disappointments. In such cases, failure can provide valuable lessons that can lead to personal growth and increased resilience.

## The Human Element

The Human Element is a generic name for describing what makes humans behave the way they do and the consequences that can have a role in the failure and errors of an event, or organization, due to poor communication, lack of motivation, cultural misunderstandings, and hidden barriers that stand in the way of new ideas.

Furthermore, it is a process for addressing and resolving human issues in the workplace and a way to convince and encourage workers to adopt new ways of doing things to reduce failures, increase performance, and boost the application of the objectives by dealing with root causes rather than shallow issues, eliminating the behaviors that sabotage and demoralize relationships and lower motivation. To accomplish these organizational changes is essential to get the support of our workers, that are often involved in other tasks. This strategy can be affected by strikes and corruption, which could be improved by the division of labor as described below. So, it can be said that the human element is closely related to failures and errors in many cases, as both can cause and contribute to problems that can ultimately lead to failures^[^6^]^.

## The Organizer

An *“organizer”* is someone who is responsible for planning, coordinating, and overseeing an event or organization by systematic planning and coordinating the effort of others^[^7^]^.They are typically in charge of tasks such as setting a budget, recruiting volunteers, and ensuring that all the necessary resources are available. Often, they work closely with other stakeholders to ensure that everything runs smoothly and goals are achieved; conversely, if the organizer does not properly train volunteers and does not effectively communicate with stakeholders, it can lead to confusion and mistakes that can ultimately cause the event or organization to fail. Something similar can happen if the organizer does not consider and anticipate the emotional reactions of the employees to a change in the organization, that can also lead to resistance and failures.

Organizers can also visualize how things may be different and are always trying to figure out the best way to reach their goals — particularly, if they dedicate substantial time to make a change, that indicates that what they are trying to modify is essential. This process is called *“vision”* that guides the energetic and committed organizers. Figuring out how to get there is known as *“strategy”,* and both are called *“theory”;* we need both to be successful. A way to find the organizers is to observe the folks already working in the organization, who should be the best candidates for finding solutions. The next step is training them!

## Division of Labor

Is a separation of tasks and responsibilities among different individuals or groups within an organization that is often used to increase efficiency and productivity. The assignment of tasks is done according to the people’s different capabilities to improve efficiency and productivity; it is the strategy to consider if you sense that your collaborators and workers are overloaded. It was published in 1776 and defined in the first sentence of “An Inquiry into the Nature and Causes of the Wealth of Nations”, the magnum opus of Adam Smith, a Scottish economist and moral philosopher^[^8^]^.

However, *division of labor* can also contribute to failures in an organization if it is not implemented or managed properly. For example, if the tasks and responsibilities are not clearly defined or communicated, it can lead to confusion and mistakes, which can result in delays, errors, and, ultimately, failure.

Additionally, if *division of labor* is not balanced, it can lead to overburdening of certain individuals or groups, which can also contribute to failures. For example, if one group is given too many tasks and responsibilities, they may become overwhelmed and unable to perform their duties effectively. This can lead to delays and errors, which can cause failures. Similarly, if there is lack of proper communication among different individuals or groups, it can result in lack of coordination and, ultimately, failures.

Overall, *division of labor* can be a powerful tool for increasing efficiency and productivity, but it is important to properly implement and manage this strategy to avoid potential failures.

## Raising the Bar by Upward Comparison

It means setting higher standards or goals for oneself or an organization by comparing them to those who are performing better or achieving more. This type of comparison can be used as a motivator to improve performance and achieve greater success. For example, an individual who is a part of a sports team may compare the team’s performance to that of a team that consistently wins championships. This comparison may motivate the players to work harder and improve their skills, with the goal of achieving the same level of success of the other team.

In an organizational context, a company may compare their productivity to that of a competitor that is consistently outperforming them. This comparison can motivate the company to raise the bar by setting higher goals and standards for themselves to improve their performance and achieve greater success.

Since we tend to overestimate where we stand in contrast to others, comparing ourselves to others is not bad. It can diminish our success — we are not the best anymore —, which can encourage us to learn. Although looking around us can be punishing, watching good players could improve our performance^[^9^, ^10^]^.

## Empathy

Empathy can be a crucial factor in reducing errors in organizational settings. When individuals or organizations are able to empathize with others, they can better anticipate and understand the needs and perspectives of those around them. This understanding can improve communication and collaboration, resulting in a reduced likelihood of errors in the administration of complex projects or tasks.

Empathy is not sympathy. Sympathy involves understanding others from your own perspective while empathy includes putting yourself in the other person’s shoes and understanding *why* they may have these feelings. Empathy can produce compassion, enabling clinicians to connect with patients and act in their best interests — studies have shown that it can also affect healthcare outcomes and even reduce the length of hospital stays.

Empathy is important because it gives you a reason to help others and to develop the kindness needed for those that require support. In addition, empathy has a snowball effect, and one empathetic person can inspire many people, which can have a positive impact. Empathy is one of the key elements of teamwork. When leaders and teammates overlook the human need for empathy, morale and performance suffers.

Demonstrating empathy in the workplace is a key part of emotional intelligence — it improves human interactions in general that can lead to more effective communication and positive outcomes. Understanding those you lead and you work with reduces barriers and resolves conflicts that otherwise block organizational success. Empathy can also be important in dealing with other stakeholders such as partners, suppliers, and investors. By being able to empathize with them, an organization may be able to better understand and address their concerns, which can help to reduce the likelihood of errors caused by misunderstandings or lack of communication.

However, it is important to note that empathy alone is not sufficient to prevent errors, other factors such as clear communication, clear instructions, proper training, and a culture of quality are also important for reducing errors. Altogether, empathy multiple positive effects.

## Final Thoughts

The authors hope that the topics discussed in the article will have a positive influence in diminishing errors and failures and in other issues affecting the efforts to improve the quality of life of those suffering the consequences of these errors. Consider yourself accepting failures gracefully as part of your life, and let them teach you to become stronger. We need to raise the bar by upward comparison to reduce failures and errors because we often overestimate where we stand. Although equating ourselves to others can diminish our success, it encourage us to learn, because it is better to watch good players to improve our performance^[^9^, ^10^]^. As any successful person will honestly admit, failure happens, and we all had our fair share of it. But from each failure, we learn two equally valuable lessons. *One,* that there was at least one reason we failed; and *two,* that we can rebound from that failure.

These are good points, but remember to look for a model that is more open, more balanced, more equitable and more beneficial to all, and do not demand a change in the organization if you cannot implement the new ideas.

“If a man is not willing to take some risk for his opinions, either his opinions are not good, or he is not good.” - Ezra Pound, a major figure in the early modernist poetry movement, renowned for his contributions both as a poet and a critic.

The journey from failure to success is not easy, but it is worth all the effort. Avoiding failures will help you succeed. But if you fail, work harder and never stop trying.

“Brave the storm, walk the path, and never regret it, letting your mind help you solve your problems.” - Bill Waterson, American Cartoonist.
